# Partial Repetition Costs are Reduced but not Eliminated with Practice

**DOI:** 10.5334/joc.230

**Published:** 2022-06-23

**Authors:** Lisa R. Fournier, Benjamin P. Richardson, Gordon D. Logan

**Affiliations:** 1 Department of Psychology, Washington State University, Pullman, Washington 99164-4820, USA; 2 Department of Psychology, Vanderbilt University, Nashville, Tennessee 37240, USA

**Keywords:** action planning, partial repetition costs, compatibility interference, event coding, feature binding, code confusion, automaticity, practice, chunking, interruptions, memory models

## Abstract

Often, we depart from an intended course of events to react to sudden situational demands (an intervening event) before resuming the originally planned action. Executing an action to an intervening event can be delayed if the features of this action plan *partly* overlap with an action plan retained in working memory (WM) compared to when they completely overlap or do not overlap. This delay is referred to as a partial repetition cost (PRC). PRCs are typically attributed to code confusion between action plans in WM. We tested this by training the component action plans extensively to reduce their reliance on WM. If PRCs are caused by code confusion within WM, then PRCs should be reduced and possibly eliminated with extensive practice. To test this, participants performed a partial repetition (PR) task after 0, 4 and 8.5 sessions of stimulus-response (S-R) training. In the PR task, participants saw two visual events. They retained an action to the first event while executing a speeded action to a second (intervening) event; afterwards, they executed the retained action. The two action plans either partly overlapped or did not overlap. Results showed that extensive (S-R and PR task) practice reduced but did not eliminate PRCs. A reduction in PRCs (code confusion) with practice is compatible with memory models that assume action events become more specific and less reliant on WM with practice. These findings merit expansions of PR tasks to other domains and broader conceptions of action plans that incorporate the formal structure of memory models.

## Introduction

To successfully interact within our environment, we must know what to do and when to do it. This involves generating action plans and coordinating their execution. Action plans are assumed to be cognitive representations of the stimulus environment, external actions, and the anticipated effects of these actions (Cattaneo, Caruana, Jezzini, & Rizzolatti, 2009; Elsner & Hommel, 2001; Frings et al., 2020; Greenwald, 1970; Hommel, 2019; Hommel, Müsseler, Aschersleben & Prinz, 2001; Jeannerod, 1997; Logan, 2018; Miller, Galanter & Pribram, 1960; Prinz, 1990; 1997; Rosenbaum, 2009; Shin, Proctor, & Capaldi, 2010). These representations can consist of “if-then” production rules such as: *If current stimulus is a green square, then immediately press center key followed by the upper key on a keypad with the left hand* (e.g., Anderson, 1992; Cohen et al., 1990; Davelaar, 2011; Logan, 1985; Miller et al., 1960; Rougier, Noelle, Braver, Cohen, & O’Reilly, 2005). Both the perception and action elements relevant to the goal (in the example above: the shape, color, hand, and key-press locations, and meaning e.g., “left-hand upper movement”) form the cognitive codes or “features” of the action plan (e.g., Davelaar, 2011; Henson, Eckstein, Waszak, Frings, & Horner, 2014; Frings et al., 2020; Hommel, 2019; Hommel et al., 2001; Jeannerod, 1997; Mattson & Fournier, 2008; Pfister & Kunde, 2013; Prinz, 1990).

Our ability to coordinate the execution of different action plans is critical for many complex, goal-directed behaviors such as sailing, cooking a meal, and performing surgery. Such coordination often requires one to depart from an intended course of events to react to sudden situational demands before resuming the originally planned action. Research shows that executing an action to an intervening event can be delayed if the features of this action plan *partly* overlap with an action plan retained in working memory (WM) compared to when they completely overlap or do not overlap (e.g., Behmer & Fournier, 2014; Fournier, Behmer & Stubblefield, 2014a; Hommel et al., 2001; Meyer & Gordon, 1985; Mattson, Fournier & Behmer, 2012; Richardson, Pfister & Fournier, 2021; Sevald & Dell, 1994; Stoet & Hommel, 1999; 2002; Yaniv, Meyer, Gordon, Huff, & Sevald, 1990). For example, executing a left-hand action can be delayed if it shares a feature (“left”) with an action plan retained in memory (“left-hand move up”) compared to when it does not (“right-hand move up”; Wiediger & Fournier, 2008). This delay is referred to as a partial repetition cost.

Partial repetition costs (PRCs) are assumed to occur when a feature code from the action plan corresponding to the intervening event (e.g., “left”) shares a feature code with the action plan retained in WM (e.g., “left hand move up”) and reactivates (primes) that action. We call this the working memory (WM) hypothesis. The action features are integrated in the action plan, so reactivating (priming) one feature code (e.g., “left” in the action plan “left hand move up”) should activate other features with which it is integrated. The reactivation of the retained action plan creates a temporary confusion as to which action plan is relevant for the current task: the action plan activated by the intervening event or the action plan recently reactivated in WM (e.g., Frings et al. 2020; Hommel, 2004, 2005; 2019; Mattson & Fournier, 2008; Mattson, Fournier, & Behmer, 2012). To resolve this confusion (code confusion), the irrelevant action plan must be inhibited, and the time required to inhibit it delays selection of the action plan for the current, intervening task (see also Fournier, Behmer, & Stubblefield, 2014a; Fournier, Hansen, Stubblefield & Van Dongen, 2020; Fournier, Gallimore, Feiszli & Logan, 2014b; Meyer & Gordon, 1985; Sevald & Dell, 1994; Yaniv, Meyer, Gordon, Huff, & Sevald, 1990).^1^ On the other hand, if there is no feature overlap between action plans, then there should be no delays in selecting the action plan for the intervening event due to such confusion. Also, if there is complete feature overlap between action plans, then the retained action plan could prime the action to the intervening event—leading to faster responding compared to when there is no overlap.

The theory that PRCs reflect competition between action plans in WM implies that PRCs should be reduced or eliminated when action plans do not rely on WM. Consistent with this hypothesis, PRCs occur if both actions rely on stimulus identity where the stimulus-response (S-R) mappings are generated offline (prior to response execution), are newly learned, and rely on WM—even if these actions do not share the same motor response (e.g., manual and vocal responses; Fournier et al., 2010). In contrast, PRCs do *not* occur if the intervening action is a reach action generated online (during action execution) based on the spatial metrics of the stimulus, without reference to stimulus identity, S-R mappings, or reliance on memory (e.g., Fournier et al., 2015; Wiediger & Fournier, 2008; see also review by Thomaschke, Hopkins & Miall, 2012) even if the actions require similar motor movements (e.g., responses with the same limb, Fournier, Wiediger, & Taddesse, 2015; Wiediger & Fournier, 2008; see also Glover, 2004; Glover, Wall & Smith, 2012). Online actions do not rely on access to WM (e.g., Goodale, 2014; Goodale & Milner, 1992) and hence would not compete with the retained action plan in terms of response selection due to common code confusion. Instead, the shared action features retained in WM could prime motor responses common with the intervening, online action, facilitating its execution (i.e., leading to a partial repetition benefit, PRB; Fournier, Wiediger & Tadesse, 2015; Wiediger & Fournier, 2008). In short, research suggests that PRCs occur if the intervening action is generated offline and relies on WM, and PRBs occur if the intervening action is generated online, and hence does not rely on WM.

To date, PRCs are typically observed for intervening actions that are newly learned and have arbitrary S-R mappings. There is some evidence that the size of PRCs may be reduced when action plans generated offline impose less of a demand on WM (e.g., word reading, Fournier et al., 2010; stimuli are ideomotor compatible, Fournier & Richardson, 2021; or individuals have higher WM spans, Fournier et al., 2014a; Behmer & Fournier, 2014). The present experiment tested the *long-term memory hypothesis*, asking whether PRCs can be eliminated for extensive practice with individual action plans. Extensive practice should increase reliance on long-term memory (LTM; Logan, 1978; 1988) and reduce reliance on WM (Logan, 1979; Shiffrin & Schneider, 1977). Less reliance on WM should reduce the opportunity for code confusion and therefore reduce the magnitude of the PRCs.

We investigated whether PRCs for actions generated offline are reduced or eliminated for action plans that are highly practiced. The long-term memory (LTM) hypothesis is consistent with association theories applied to skill learning and memory (e.g., ACT, Anderson, 1992; Instance theory, Logan, 1988) and feature integration theories applied to perception (Treisman & Gelade, 1980). We assumed the following: Novel action plans (i.e., unpracticed, newly learned, with arbitrary S-R mappings) have weak associations and require WM to maintain feature bindings and S-R rules (see also Davelaar, 2011). Thus, both the retained and intervening action plans would need to utilize WM at the same time and hence would be susceptible to code confusion. Practiced, more familiar action plans (with arbitrary S-R mappings) have stronger associations and impose less of a demand on WM to maintain them (e.g., Logan, 1979; Posner & Snyder, 1975; Shiffrin & Schneider, 1977). Over extensive practice, action plans (with arbitrary S-R mappings) transition from WM to LTM (e.g., Anderson, 1982; 1992; Anderson, Fincham, & Douglass, 1999; Jueptner et al., 1997; Logan, 1988; Posner & Snyder, 1975; Shiffrin & Schneider, 1977). When association strength is sufficiently high, each action plan can be retrieved directly from LTM as a single, integrated representation (e.g., Anderson, 1992; Anderson et al., 1999; Fitts & Posner, 1967; Logan, 1979, 2018; Logan & Etherton, 1994; Newell & Rosenbloom, 1981; Rosenbloom & Newell, 1986; Sakai, Kitaguchi, & Hikosaka, 2003; Verwey, 1999; Woltz, 1988). As a result, the retained and intervening action plans do not need to be represented in WM at the same time and hence would *not* be susceptible to code confusion.

Anderson’s (1982) production system approach to skill acquisition provides another concrete example. Novel action plans are represented by binding stimulus events to conditions and actions of generic production rules that allow the task to be executed. The bindings are held in WM and may compete when they are both activated by a common component of their action plans. With practice, performance shifts from declarative representations in WM to procedural representations in LTM. The stimuli relevant to the action plan become embedded in the condition (“if” part) of the production and do not need to be bound in WM to be executed. When the action is executed, there is no confusion about which codes are bound in WM and so, the PRC is reduced or eliminated.

Our hypothesis that automatized action plans may not produce PRCs receives some support in studies of skilled typewriting that present prime words followed by probes to be typed. Some probes required the letters that were bound to the prime word to be typed in a new context. These probes should show PRCs relative to controls. Crump and Logan (2010) presented word primes followed by a repetition of the same word, a single letter from the word (partial repetition), or a single letter from another word. Skilled typists typed probe letters from the prime word more quickly than probe letters from another word, showing a partial repetition benefit instead of a cost. Snyder and Logan (2014) presented prime words (e.g., OCEAN) followed by a go signal (*****) or a word to type. The probe word was either identical to the prime (OCEAN), an anagram of the prime (CANOE), sharing common letters, or different from the prime, sharing no letters with it (GULPS). Anagram primes were no slower than different primes. There was no PRC. These results replicated in subsequent experiments with probes that consisted of a mixture of letters that did and did not appear in the prime (e.g., SSDD, DDSS, SDDS, DSSD, SDDD, SSSD where S = same as prime and D = different from prime). There was no PRC (compared to all different letters) in any experiment. The typists were well practiced, having typed for about 10 years and spending about 4 hours typing each day (Logan & Crump, 2011), so their motor plans were highly automatic (Logan, 2018). By our hypothesis, they should not show PRCs.

We used the partial repetition (PR) task (e.g., Fournier et al., 2014a, b; Stoet & Hommel, 1999) to investigate whether partial repetition costs found for novel action plans (newly learned, arbitrary S-R mappings) are reduced or eliminated with extensive practice. Participants saw two, visual events presented in a sequence. At the presentation of the first event (event A), a response to event A was planned and retained in WM. During this retention period, the second event (event B) appeared, and a speeded response was made to this intervening event. Afterwards, the retained action plan to event A was recalled and executed. For both events A and B, the stimuli and response mappings were arbitrary (e.g., a letter or symbol mapped to a series of different key-press responses, executed by the left or right hand). We manipulated action overlap between event A and B by requiring the same hand (overlap) or different hands (no overlap) to execute a series of key-press responses. We also manipulated the amount of S-R training for event A and event B stimuli and examined the PRC effect with no S-R practice, four sessions of S-R practice (1200 and 600 trials for each event B and event A stimulus, respectively), and eight and a half sessions of S-R practice (2460 and 1230 trials for each event B and event A stimulus, respectively).

Consistent with the WM hypothesis, we expected to find PRCs when the intervening action (to event B) and the retained action (to event A) were not practiced (PR session 1). In this case, both actions would be represented in WM, so code confusion can occur, which would delay responses to the intervening event. Consistent with the LTM hypothesis, we expected that the PRCs initially observed prior to S-R practice (PR session 1) would be significantly reduced or eliminated after extensive S-R practice with event A and event B stimuli (PR session 3).

## Method

### Participants

Fifteen students from Washington State University and one non-student, resident of Pullman, Washington participated for monetary compensation. One participant’s data were incomplete due to a program error, and hence data were analyzed for 14 participants (mean age 25.6 years, standard deviation 5.8 years, all right-handed, 11 female). Effect sizes for partial repetition costs were reported to be large in several previous studies (e.g., {d_z} = \frac{{\sqrt F }}{{\sqrt n }} = \frac{{\sqrt {37.31} }}{{\sqrt {59} }} = 0.80 for the critical pairwise comparison of overlap and no-overlap trials in Fournier et al. (2014a), and {d_z} = \frac{{\sqrt {18.42} }}{{\sqrt {18} }} = 1.01 for the same comparison in Experiment 1 of Stoet & Hommel (1999). Assuming that PRCs come with a large effect size of *d*_z_ = 0.80, a power analysis suggested a sample size of eight participants would be necessary to detect a significant interaction between the within-subjects factors of action overlap (overlap and no overlap) and session (i.e., first, middle, and last PR task session: session 1, 6 and 11), with a power of *1–β* = .80 and an effect size of .60. To ensure sufficient power, we planned to collect data from 14 total participants, a priori. This study was approved by the Washington State University Institutional Review Board (IRB, 15378), and informed consent was obtained from all participants.

### Apparatus

Visual stimuli appeared in white, Arial font with a black background on a 17” CRT monitor, located approximately 52 cm in front of the participant. Responses were recorded using a custom response keypad (X-Keys XK80 USB Keyboard, Williamston, MI), placed on a desk, centered at the participant’s midline. Participants responded using three vertically aligned keys located at the bottom left and at the bottom right of the keypad. The horizontal separation between the left and right response keys (center-to-center) was 20 cm. The immediately surrounding keys were blocked from access with rigid, black key caps. Responses were made with the index fingers of the left and right hands; left-hand responses were executed on the left side of the keypad and right-hand responses on the right side. Participants rested their index fingers on the left and right center keys before and during each trial. The participant’s hands and keypad were visible when looking down. E-Prime software (version 2.0.10.356; Psychology Software Tools, Inc., Sharpsburg, PA) was used for stimulus generation, stimulus presentation, and data collection.

### Stimulus events and response mappings

#### Event A

Event A was one of five arrowhead stimuli appearing (0.17° visual angle) above a central fixation dot. One stimulus consisted of two arrowheads pointing outward (< >) and the other four consisted of an arrowhead (0.67° visual angle) pointing to the left (<) or right (>) with an asterisk (*; 0.45° visual angle) above or below the arrowhead. For the stimuli composed of two arrowheads pointing outward (< >), no response was required. For all other stimuli, the arrowhead direction (left or right) indicated the response hand (left or right, respectively) and the asterisk’s location (above or below the arrowhead) indicated the three, keypress sequence. The asterisk located *above* the arrowhead required a “center key, *upper* key (toward CRT), and center key” response and the asterisk located *below* the arrowhead required a “center key, *lower* key (toward participant’s body), and center key” response. This created four different action plans mapped to the arrowhead-asterisk stimuli: *left hand-move up, left hand-move down, right hand-move up*, and *right hand-move down*. The four event A stimuli and their corresponding responses are shown in Table 1.

**Table 1:** Stimulus types and associated response mappings for each event A and event B stimulus, respectively.


	EVENT A				EVENT B	
	
STIMULUS	* <	< *	* >	> *	C	S

Response Hand	Left	Left	Right	Right	Left	Right

Keys	Center Up Center	Center Down Center	Center Up Center	Center Down Center	Center Center	Center Center


#### Event B

Event B was one of two capitalized letters, C or S (0.79° visual angle), superimposed on a central fixation dot (shifted slightly below center of the dot, so that the dot was visible). The letters C and S were consistently mapped to a left or right response. Half the participants pressed the left center key twice with their left index finger in response to the letter C and pressed the right center key twice with their right index finger in response to the letter S; the other half had the opposite stimulus-response mapping. See Table 1. Event B required two presses of the center key, so that action B and action A represented two distinctly different sequences of keypresses (see Stoet & Hommel, 1999).

### Procedure

**Figure 1:**
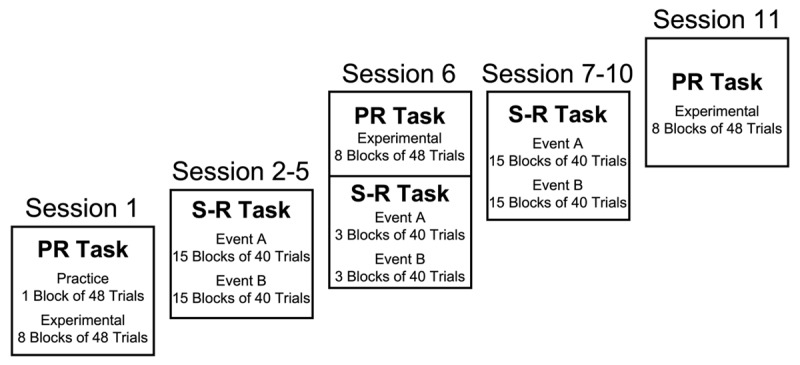
The figure shows the order of PR tasks and S-R training tasks across sessions with a breakdown of blocks for each task. The amount of practice for each S-R mapping was as follows: Session 1 represented no previous S-R practice, session 6 represented a moderate amount of S-R practice (1200 trials for each event B stimulus, S and C; 600 trials for each event A stimulus, 4 different arrowhead-asterisk combinations), and session 11 represented extensive S-R practice (2460 trials for each event B stimulus, S and C; and 1230 total trials for each event A stimulus, 4 different arrowhead-asterisk combinations).

Participants completed 11 sessions, one session (60–90 minutes) per day, within 15 days. Participants performed the partial repetition (PR) task in sessions 1, 6, and 11, and performed the stimulus-response (S-R) training task in sessions 2–10. The PR and S-R training tasks occurred on separate days except for session 6; here, the PR task was performed first followed by a shortened, S-R training task. Figure 1 shows the order of PR and S-R training tasks across sessions along with the breakdown of trial blocks for the tasks in each session. For both tasks, the instructions (self-paced) appeared on the CRT, and stimulus-response mappings were identical. Participants completed a 10-question strategy survey at end of session 11.

### PR task

Participants saw two stimulus events (A and B) in a sequence. They were instructed as follows: Plan an action (action A) to the first event (A) and retain this action in memory while executing an immediate action (action B) to the second event (B) as quickly and accurately as possible. After executing action B, recall and execute the action A as accurately as possible. If the first event (A) does not require an action (i.e., the stimulus was “< >”), do not plan or retain an action to this event. Also, do not execute any part of action A until after executing action B. Finally, do not move fingers or use external cues to help remember action A –maintain action A in memory.^2^

**Figure 2:**
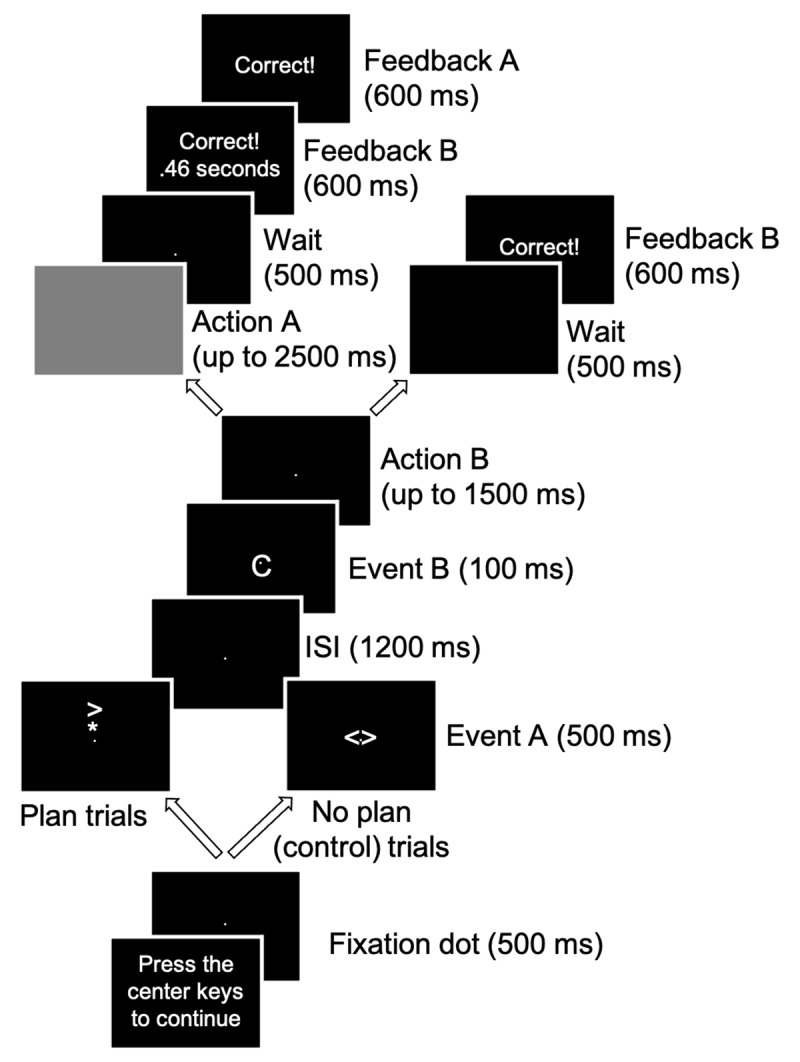
The sequence of trial events for the partial repetition (PR) task. The frames in the center and on the left represent the trials in which participants planned and retained an action for event A (plan trials). The frames in the center and on the right represent the trials in which no action was planned to event A (no plan, control trials).

Figure 2 shows the sequence and timing of trial events for the PR task. Each trial began with an initiation screen that read “Press the center keys to continue”. After pressing these keys simultaneously, a central fixation dot appeared for 500 ms, followed by event A for 500 ms, and then the fixation dot alone for 1200 ms. During the appearance of event A and the fixation that followed, participants planned the action for event A (if applicable). Then, event B appeared for 100 ms, followed by the fixation dot alone until action B was executed or 2500 ms elapsed. Action B RT was recorded from the onset of event B to the first key-press response. After executing action B, a blank screen appeared for a maximum of 2000 ms. During this time, participants executed action A (if applicable) or waited. Afterwards, performance feedback for action B (RT and accuracy) and action A (accuracy only) appeared respectively, for 600 ms each. Then, the initiation screen re-appeared, and participants initiated the next trial when ready.

Two factors were manipulated within participants: action overlap and session. For action overlap, action B and action A either required the same hand (overlap) or different hands (no overlap). The condition in which event A did not require a response (< >) was not analyzed, as this condition served only as a control to ensure that any lack of differences in action B responses found between overlap and no overlap conditions could not be attributed to a floor effect in RTs. The factor of session represented three different levels of S-R practice for both event B and event A stimuli: session 1 represented no previous S-R practice, session 6 represented a moderate amount of S-R practice (1200 trials for each event B stimulus, S and C; 600 trials for each event A stimulus, 4 different arrowhead-asterisk combinations), and session 11 represented extensive S-R practice (2460 trials for each event B stimulus, S and C; and 1230 total trials for each event A stimulus, 4 different arrowhead-asterisk combinations).

The PR task required approximately 60 min. Participants completed eight experimental blocks of 48 trials with mandatory 10 sec breaks at the end of each block. Within each block, there were 16 overlap, 16 no overlap, and 16 no plan (control) trials. Also, event B stimuli were equally paired with event A stimuli and event pairs occurred in a random order within each block. In session 1, participants received one block of practice trials identical to the experimental blocks.

### S-R Training

**Figure 3:**
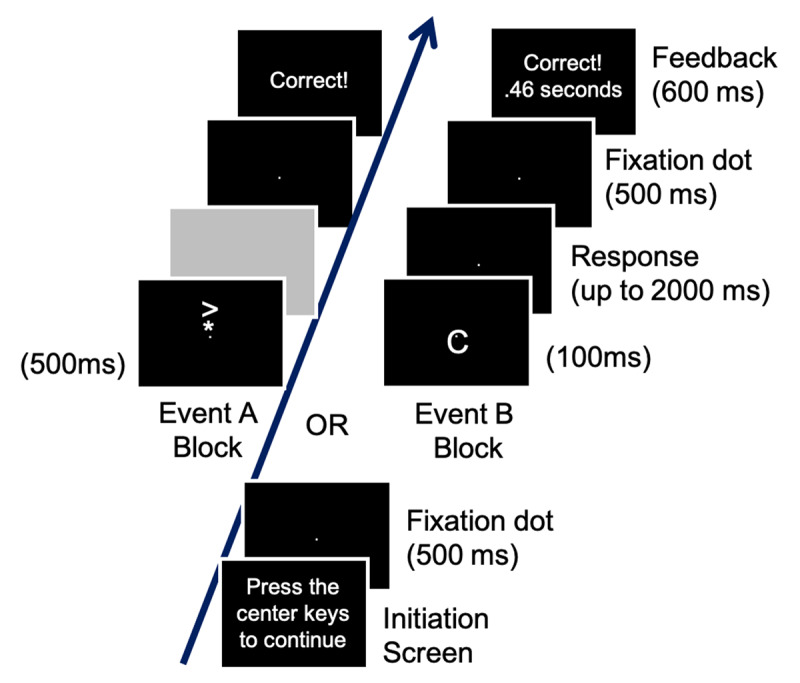
The sequence of trial events for the S-R training task. The sequence for the event A stimulus blocks is shown in the central and left frames. The sequence for the event B stimulus blocks is shown in the central and right frames.

Participants received S-R training for event A stimuli and event B stimuli in separate, alternating blocks within and across sessions. Figure 3 shows an example of the sequence of events for training trials for both event A and event B stimulus blocks. Each block started with a message indicating which type of stimuli (event A or event B) would be presented. Trials always started with a message “Press the center keys to continue” (initiation screen). Participants started each trial by simultaneously pressing the center keys with their index fingers. Then, 500 ms later, a stimulus appeared. For event A stimulus blocks, stimulus A was present for 500 ms; for event B stimulus blocks, stimulus B was present for 100 ms. Participants had 2000 ms to execute the correct key-press response. Participants were encouraged to respond as quickly and accurately as possible. Performance feedback (RT and accuracy for event B blocks and accuracy only for event A blocks) appeared 500 ms after their last key-press response for a duration of 600 ms. Then, the initiation screen re-appeared, and participants started the next trial when ready. We recorded response RT (from onset of the stimulus to onset of the first key-press response for event B stimuli and to the last key-press response for event A stimuli) and accuracy (to all required keypresses) for each trial.

Participants completed 30 blocks of 40 trials of S-R training (15 blocks each for event A and event B) in sessions 2–5 and 7–10, and 6 blocks of 40 trials (3 blocks each for event A and event B) in session 6. The specific number of training trials for each stimulus within the event A stimulus set and event B stimulus set prior to each PR task session is reported under manipulations of the PR task (see above) and in the caption for Figure 1.

## Results

### S-R training task

To test the LTM hypothesis that extensive practice with S-R mappings reduces or eliminates the PRC effect, we must first demonstrate that practice on S-R mapping improves performance. Analyses of both RTs and error rates provide evidence that S-R training led to more automatic retrieval of action plans (action B and action A) associated with event B and event A stimulus sets. Separate, repeated-measures ANOVAs with the factor of session (2–10) were conducted on median correct RTs and mean error rates for each action B response (2 types: left and right) and each action A response (4 types: left up, left down, right up, and right down). Action B correct RTs were evaluated based on the first key-press response, and action A correct RTs were evaluated based on the third key-press response (i.e., the first response to action A). Responses were considered accurate only if all required key-press responses were executed in the correct order. For each type of action B and action A response, there was a significant effect of session found for RTs; no session effects were found for error rates with the exception of the action A response of “right up”. The ANOVA results for each response type are presented in Table 2.

**Table 2:** S-R training trials: One-way, repeated-measures ANOVAs with the factor of training session were conducted separately on response reaction times (RT) and error rates for each action B and action A response type.


RESPONSE TYPE	FACTOR	SIGNIFICANCE	*ηP^2^*

*MEDIAN CORRECT RT*			

action B, left	Session	*F*(8, 104) = 6.20, *p <* .001	0.32

action B, right	Session	*F*(8, 104) = 5.22, *p* = .001	0.29

action A, left up	Session	*F*(8, 104) = 15.22, *p* < .001	0.54

action A, left down	Session	*F*(8, 104) = 20.14, *p* < .001	0.61

action A, right up	Session	*F*(8, 104) = 14.20, *p* < .001	0.53

action A, right down	Session	*F*(8, 104) = 17.20, *p* < .001	0.57

***MEAN ERROR RATES***			

action B, left	Session	*F*(8, 104) = 1.61, *p* = .208	0.11

action B, right	Session	*F*(8, 104) = 2.45, *p* = .07	0.16

action A, left up	Session	*F*(8, 104) = 1.78, *p* = .09	0.12

action A, left down	Session	*F* < 1	

action A, right up	Session	*F*(8, 104) = 2.19, *p* = .034	0.14

action A, right down	Session	*F* < 1	


### Correct RTs

**Figure 4:**
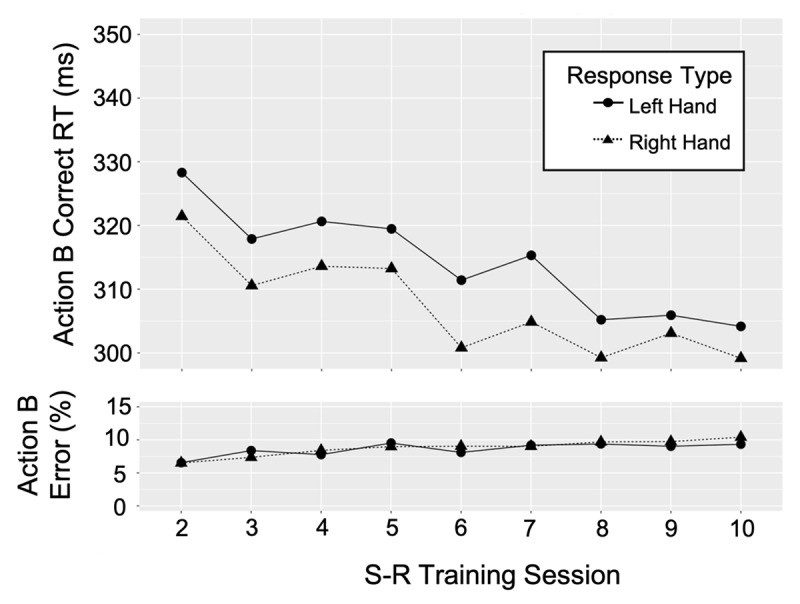
Action B, median correct RTs (first, key-press response) and percent error rates for each response type in each S-R training task session.

**Figure 5:**
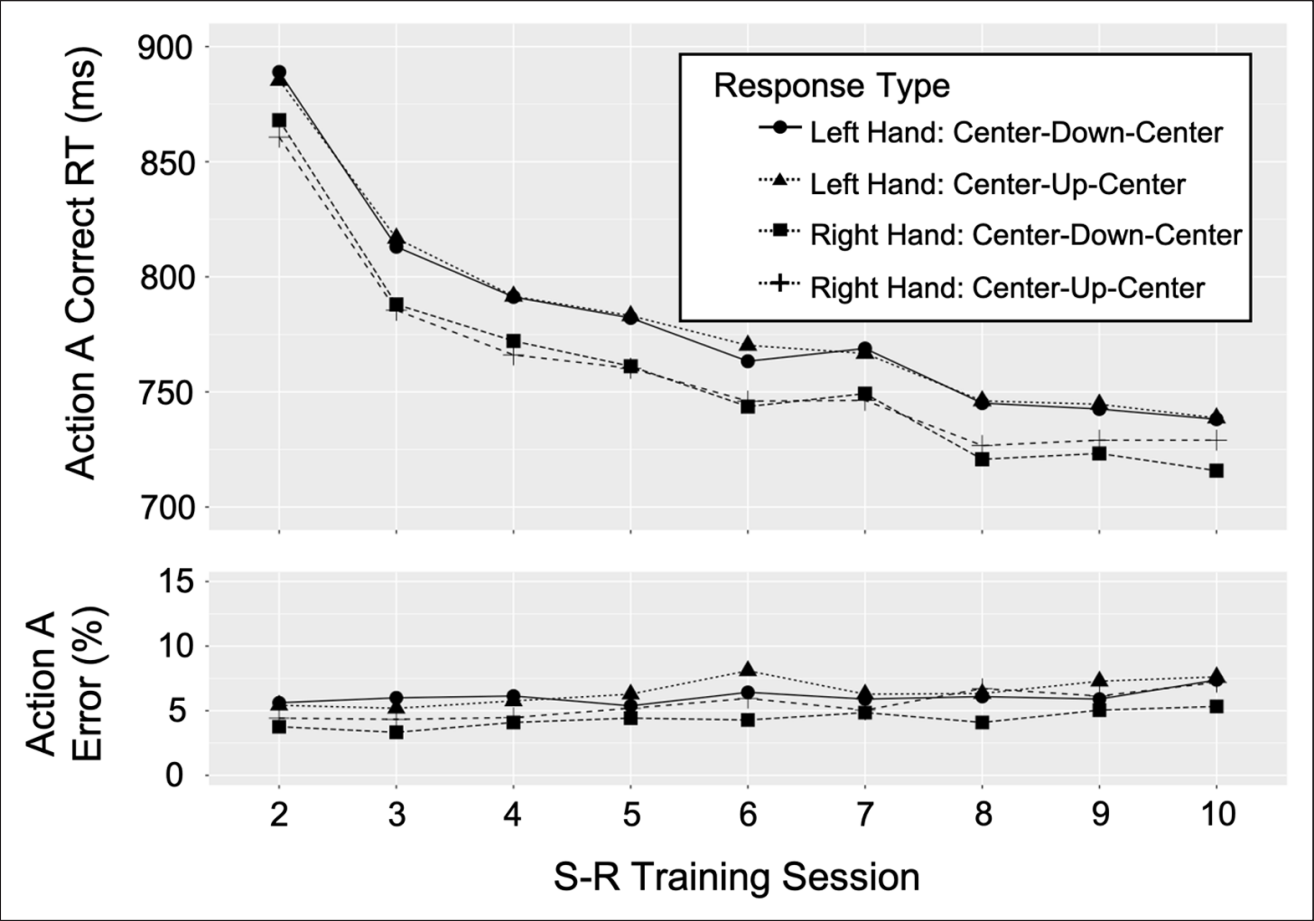
Action A, median correct RTs (third, key-press response) and percent error rates for each response type in each S-R training task session.

Figures 4 and 5 show the median correct RTs for the first key-press response to event B stimuli (action B) and third key-press response to event A stimuli (action A), respectively, for each hand and key-press sequence by S-R training session. Both figures show a speed up in correct RTs across sessions which is consistent with the power law of learning (e.g., Logan, 1988). Paired *t* tests assessed whether RTs for each action B response and each action A response achieved asymptote toward the end of the training sessions (See Appendix A). If three or more sessions, starting with the last session (session 10) did not significantly differ, and the average RT across these sessions were shown to be significantly faster than any prior session (session 7, 6, 5, 4, etc.), asymptote was assumed. Results reported in Appendix A suggest that all correct RTs for action B and action A responses were at asymptote in sessions 8–10. Thus, these findings indicate that retrieval of action B and action A were more automatic prior to performing the final session of the PR task (session 11) compared to the first (session 1) and second (session 6) sessions of the PR task. This suggests that action control was transferred from WM to LTM, consistent with the LTM hypothesis.

#### Error Rates

No significant effects of session were found for action B or action A error rates except for the action A response of “right up”. For these responses, error rates increased across sessions (session 1 = 4.36% and session 8 = 7.21%) suggesting that the correct RTs across sessions for this response (but not the other action A responses) may be partly attributed to a speed-accuracy tradeoff. Importantly, the action B error rates do not compromise any action B RT interpretations above.

### PR Task

Action B correct RT and error rate analyses were restricted to trials in which action A was accurate. Also, action B correct RTs represent the interval between stimulus B onset to the *first* key-press for trials in which both action B key-presses were accurately executed. Action A error rates were analyzed without a contingency on action B accuracy.^3^

### Action B mean response analyses

**Figure 6:**
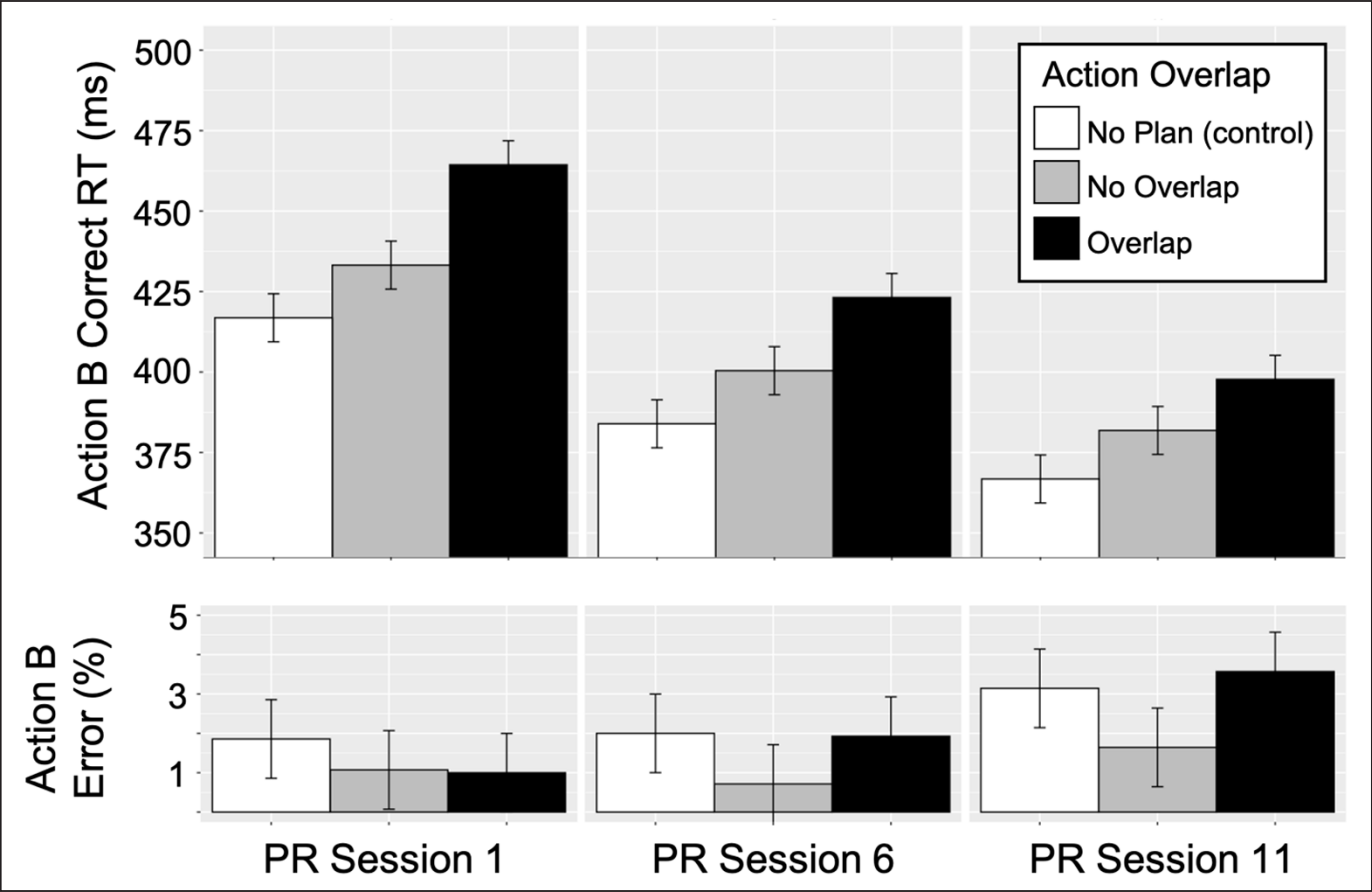
Action B mean correct RT (first key-press response) and action B percent errors for the action overlap conditions (no overlap and overlap) and the no plan (control) condition by each partial repetition (PR) task session (1, 6, and 11). Error bars represent the within-subjects standard errors (Loftus & Mason, 1994).

Repeated-measures ANOVAs with the factors of action overlap (no overlap, overlap) and session (1, 6, 11) were conducted separately on means for action B correct RTs and error rates. Figure 6 shows the mean, action B correct RTs and error rates for the action overlap conditions across the three, PR task sessions. The no planning (control) condition is represented in the figure to show that small RT differences between overlap and no overlap conditions (i.e., PRCs) were not due to a floor effect.

#### Mean correct RT

As expected, RTs were greater for the overlap compared to the no overlap condition indicating a partial repetition cost (PRC) [main effect of action overlap, *F*(1, 13) = 26.59, *p* < .001, *ηp^2^* = 0.67)]. Also as expected, RTs decreased for both action overlap conditions across session [main effect of session, *F*(2, 26) = 28.02, *p* < .001, *ηp^2^* = 0.68)]. Moreover, the interaction was significant [*F*(2, 26) = 6.26, *p* = .006, *ηp^2^* = 0.32)] indicating that the RT differences between the overlap and no overlap conditions (PRCs) were significantly reduced across sessions. See Figure 6. The size of PRCs was approximately 33, 23, and 16 ms for session 1, 6, and 11 respectively. Planned comparisons showed that the size of the PRC was significantly reduced in session 11 compared to session 1 [*t*(13) = 2.50, *p* = .027, *d* = .67]^4^ but the PRC was not eliminated. PRCs were significant for each session [session 1, *t*(13) = 5.47, *p* < .001, *d* = 1.46; session 6, *t*(13) = 4.06, *p* = .001, *d* = 1.09; and session 11, *t*(13) = 3.68, *p* = .003, *d* = 0.98].

#### Mean error rates

For error rates, there were no significant effects of action overlap [*F*(1, 13) = 3.20, *p* = .097)], session [*F*(2, 26) = 1.12, *p* = .312], or the interaction [*F*(2, 26) = 2.65, *p* = .090]. Figure 6 shows that overall error rates were less than 5% across sessions. The trend toward higher error rates for the overlap compared to the no overlap condition is consistent with the PRCs reported for correct RTs.

### Action A error rates

A repeated-measures ANOVA with the factors of action overlap (no overlap, overlap) and session (1, 6, 11) was conducted on action A error rates. Table 3 reports the error rates for the no overlap and overlap conditions by session. Error rates were greater for the overlap (5.93%) compared to the no overlap (4.60%) condition [main effect of action overlap, *F*(1, 13) = 5.11, *p* = .042, *ηp^2^* = 0.28], consistent with previous studies (e.g., Fournier, Behmer, et al., 2014; Richardson et al., 2020). No significant effects of practice were found (main effect of session and the interaction, *Fs* < 1, respectively). Importantly, error rates were low overall, indicating that participants were generally able to accurately recall the retained action.

**Table 3:** Mean recall error rates and within-subject standard errors (SEs) for the retained action plan (action A) by PR-task session and action overlap condition.


PR-TASK SESSION	ACTION OVERLAP	MEAN ERROR %	SE %

1	Overlap	6.79	1.15

	No Overlap	5.21	1.01

6	Overlap	5.57	1.10

	No Overlap	4.57	0.94

11	Overlap	5.43	1.06

	No Overlap	4.00	1.12


## Discussion

We found that partial repetition costs (PRCs) were reduced but not eliminated when responses corresponding to specific perceptual events were highly practiced (i.e., eight and a half sessions of S-R practice; 2460 and 1230 trials for each event B and event A stimulus, respectively). The results early in practice are consistent with the WM hypothesis, which says that PRCs result from code confusion between action plans held in WM. The results late in practice are consistent with the LTM hypothesis, which says that extensively practiced action plans can be retrieved directly from LTM (Logan, 1988, 2018). We attribute the reduction in the size of PRCs across PR task sessions to extended practice on the S-R training task, but it could (also) be due to practice on the PR task, or practice on both tasks. However, there were only two sessions of practice on the PR task (384 and 128 trials for each event B and event A stimulus, respectively) compared to eight and a half sessions with each S-R task (2460 and 1230 trials for each event B and event A stimulus, respectively), so S-R training may have contributed more to the results.

We assumed that with extensive practice, intervening action plans would no longer rely on WM and instead would be directly retrieved from LTM. Our results showed that correct responses for the intervening action became faster across the three PR task sessions suggesting that the action plan retrieval became more efficient over time—consistent with a decrease in WM demands. However, PRCs were not eliminated with extensive practice, which suggests that code confusion between action plans persisted in our study. Thus, extensive practice did not allow the representations of the retained and intervening actions to rely entirely on LTM and so avoid code confusion. It is unclear whether more practice (such as the amount of practice in our typing example; Crump & Logan, 2010; Snyder & Logan, 2014) would have eliminated code confusion. Importantly, however, the reduction of code confusion with practice found in our study is compatible with several memory models that explain improvements in performance with practice.

Anderson’s (1982) ACT theory specifically predicts that practice produces a transition from performance based on declarative WM to procedural LTM. Novel tasks are performed by binding relevant stimuli and responses to generic production rules that represent the instructions declaratively. Through *proceduralization*, the stimuli and responses become embedded directly in the condition and action terms of task-specific productions, so they no longer require binding in WM. Thus, PRCs should occur early in practice but not late in practice. In ACT, reliance on procedural and declarative memory is strategic. The persisting PRCs in the last practice session may reflect a reluctance to abandon the WM strategy entirely and trust LTM retrieval to do the task.

More generally, our results suggest that chunking of features stored in LTM within or across action events could contribute to a reduction in code confusion with practice (Logan, 2018). Chunking could reduce confusion between action events and improve retrieval by integrating contextual cues making each chunked action event more distinct from other chunks (e.g., Yamaguchi & Logan, 2016; Yamaguchi, Randle, Wilson & Logan, 2016) or by transforming the original feature codes representing each action event into a new representation (e.g., Anderson, 1983; Fitts & Posner, 1967). Eventually, with sufficient practice (more extensive practice than in the current study) the individually chunked action events could become sufficiently distinct such that two different chunks would rarely be confusable (but see Schneider & Logan 2015 regarding chunk boundaries). Chunking could also occur across the different action event pairings (retained and intervening event pairs) due to training in the PR task. As the retained and intervening action events are being transformed into one, integrated chunk, code confusion would be reduced and eventually eliminated as there would no longer be two distinct action events that could partly overlap and create code confusion. In sum, chunking predicts that code confusion should be reduced and perhaps eliminated if there is sufficient practice. It could be argued that more extensive training in the current study would have eliminated code confusion due to chunking within and/or across action events.

Memory models that do not rely on chunking and instead assume that action events become more differentiated or distinct with practice, can also account for the reduction in code confusion with extensive practice. For these models, feature codes that are distinct between the two action events become more salient (e.g., weighted more heavily) with practice. This weighting of distinct features would be expected to occur during PR task training but could also occur during S-R task training. For example, a code (e.g., “up”) associated with the retained action (e.g., “left up”) that is not shared with the intervening action (e.g., “left”) may become more salient or weighted more heavily with experience which would allow the two action plans (e.g., “left up” and “left”) to be more quickly distinguished. Also, the context (e.g., Logan, 2018; Polyn, Norman & Kahana, 2009) with which the retained and intervening events occur (e.g., retained action event always occurs first, intervening action always occurs second) in the PR task could be encoded as well. This could provide a further distinction among feature codes between the two action events with continued practice within the same context [e.g., temporal code associated with first event (e.g., “first=left up”) differentiates this event and its associated features from that of the second event (e.g., “second=left”)]. Here, both the temporal code of “first” and the response code of “up” could distinguish the retained event (“first=left up”) from the intervening, event (e.g., “second=left”). Thus, extended practice would lead to an overall reduction in the size of code confusion, as code confusion could be resolved more quickly and hence result in a smaller PRC.

This account for why PRCs were reduced in our study is consistent with memory models that assume each instance of an item contributes to a single growing memory trace that becomes enriched or differentiated with each additional encounter (p. 37, Cox & Criss, 2020; e.g., Kilic, Criss, Malmberg & Shiffrin, 2017; McClelland & Chappell, 1998; Shiffrin, Ratcliff, & Clark, 1990; Shiffrin & Steyvers, 1997; see Cox & Criss, 2020 for an overview). It is also consistent with memory models that assume multiple traces of item instances are stored as separate traces (Hintzman, 1988; Logan, 1988, 2002, 2018; Rosenbaum, Loukopoulos, Meulenbroek, Vaughan, & Engelbrecht, 1995; Rosenbaum, Meulenbroek, Vaughan & Jansen, 2001). Each additional encounter amplifies the similarity among instances of the same task and the dissimilarity between instances of different tasks.

While we provide evidence that code confusion for offline actions can be reduced with practice, we cannot conclude that it can be eliminated with more extensive practice. The typing data suggest it can be eliminated (Crump & Logan, 2010; Snyder & Logan, 2014), but the tasks were different from the usual PRC procedures, so some uncertainty remains. However, our results make it clear that practice is an important modulator of PRCs and that motivates further research into practice effects. Our findings are consistent with memory models that assume action events become more specific and less reliant on WM with practice. Our interpretations of the reduction of the PRC effect in terms of memory models provide new perspectives on the PRC effect and that motivates expansions of the PR tasks to other domains and broader conceptions of action plans that incorporate the formal structure of the memory models.

## Data Accessibility Statement

Data is accessible at the following repository: http://doi.org/10.7273/000001465.

## Footnotes

Whether inhibition occurs at a local feature level or central level is debated (See Fournier et al., 2014b; Fournier et al., 2020; Hommel, Proctor, & Vu, 2004; Kühn, Keizer, Colzato, Rombouts, & Hommel, 2011).

One participant violated these instructions.

Assessing response accuracy to event A was necessary to ensure participants retained the action plan to the first event in memory while executing their response to the interruption (event B). These results for event A have to be interpreted with caution as participant inclusion required that they achieve 80% accuracy. Also, we did not analyze RT for event A for two reasons. First, we told participants that we were not concerned with how fast they responded to event A, only how accurate they responded. Second, RT for event A was confounded with responses executed to event B. That is, when there was partial overlap (i.e., responses to events A and B shared the same response hand), the motor response for event A had to wait for the response to event B to finish before it could start; but when there was no overlap, the motor response for event A did not necessarily have to wait for the motor response to event B to finish before it could start.

Correct RT, cumulative probability density functions (CDFs; bin size of 1 ms) for the overlap and no overlap conditions across PR task sessions is available in Appendix B.

## Appendix A

S-R Training. Paired *t* tests suggested that all correct RTs for action B and action A were at asymptote in sessions 8–10. For action B responses (see Figure 4), left-hand key-press RTs appeared to asymptote by session 8 [*t*(13) ≤ 1.36, *p* ≥ .198 for sessions 8–10; and *t*(13) = 3.33, *p* = .005 for session 7 vs. average among sessions 8–10], and right-hand key-press responses appeared to asymptote by session 6 [*t*(13) ≤ 0.64, *p* ≥ .146 for sessions 6–10; and *t*(13) = 2.76, *p* = .016 for session 5 vs. average of sessions 6–10]. For action A (see Figure 5), “left hand down” responses appeared to asymptote by session 8 [*t*(13) ≤ 0.53, *p* ≥ .229 for sessions 8–10; and *t*(13) = 4.89, *p* < .001 for session 7 vs. average among sessions 8–10], “left hand up” responses appeared to asymptote by session 8 [*t*(13) ≤ 0.13, *p* ≥ .333 for sessions 8–10; and *t*(13) = 3.58, *p* = .003 for session 7 vs. average among sessions 8–10], “right hand down” responses appeared to asymptote by session 8 [*t*(13) ≤ 0.17, *p* ≥ .065 for sessions 8–10; and *t*(13) = 4.21, *p* = .001 for session 7 vs. average among sessions 8–10] and “right hand up” responses appeared to asymptote by session 8 [*t*(13) ≤ 0.11, *p* ≥ .671 for sessions 8–10; and *t*(13) = 3.84, *p* = .002 for session 7 vs. average among sessions 8–10].

## Appendix B

Cumulative probability density functions (CDFs) for correct action B RTs (first key press) for the overlap (black solid line), no overlap (black dotted line) and no plan (control; gray dashed line) condition for each partial repetition (PR) task session (1, 6, and 11). Correct RT bin size was 1 ms.
